# An experimental evaluation of the antidiabetic and antilipidemic properties of a standardized Momordica charantia fruit extract

**DOI:** 10.1186/1472-6882-7-29

**Published:** 2007-09-24

**Authors:** Nafisa PC Fernandes, Chakradhar V Lagishetty, Vandana S Panda, Suresh R Naik

**Affiliations:** 1 Prin.K.M.Kundnani College of Pharm, Department of Pharmacology and Toxicology, Jote Joy Building, Rambhau Salgaonkar Marg, Cuffe Parade, Mumbai 4000 05, India; 2 Sinhgad Institute of Pharmaceutical Sciences, Sector No. 309/310, Kusgaon(Bk) Lonavala, Pune 410 401, India

## Abstract

**Background:**

The MCE, *Momordica charantia fruit extract *Linn. (Cucurbitaceae) have been documented to elicit hypoglycemic activity on various occasions. However, due to lack of standardization of these extracts, their efficacy remains questionable. The present study was undertaken by selecting a well standardised MCE. This study reports hypoglycemic and antilipidemic activities of MCE employing relevant animal models and *in vitro *methods.

**Methods:**

Diabetes was induced in Wistar rats by a s.c., subcutaneous injection of alloxan monohydrate (100 mg/kg) in acetate buffer (pH 4.5). MCE and glibenclamide were administered orally to alloxan diabetic rats at doses of 150 mg/kg, 300 mg/kg & 600 mg/kg, and 4 mg/kg respectively for 30 days, blood was withdrawn for glucose determination on 0, 7, 14, 21 and 30^th ^days. On the 31^st ^day, overnight fasted rats were sacrificed and blood was collected for various biochemical estimations including glycosylated haemoglobin, mean blood glucose, serum insulin, cholesterol, triglcerides, protein and glycogen content of liver. The hemidiaphragms and livers were also isolated, carefully excised and placed immediately in ice cooled perfusion solution and processed to study the glucose uptake/transfer processes. Hypolipidemic activity in old obese rats was evaluated by treating two groups with MCE (150 mg/kg & 300 mg/kg) orally for 30 days and determining total cholesterol, triglyceride and HDL-CH, LDL-CH and VLDL-CH levels from serum samples.

**Results:**

Subchronic study of MCE in alloxan induced diabetic rats showed significant antihyperglycemic activity by lowering blood glucose and GHb%, percent glycosylated haemoglobin. Pattern of glucose tolerance curve was also altered significantly. MCE treatment enhanced uptake of glucose by hemidiaphragm and inhibited glycogenolysis in liver slices *in vitro*. A significant reduction in the serum cholesterol and glyceride levels of obese rats following MCE treatment was also observed.

**Conclusion:**

Our experimental findings with respect to the mechanism of action of MCE in alloxan diabetic rats suggest that it enhances insulin secretion by the islets of Langerhans, reduces glycogenesis in liver tissue, enhances peripheral glucose utilisation and increases serum protein levels. Furthermore, MCE treatment restores the altered histological architecture of the islets of Langerhans. Hence, the biochemical, pharmacological and histopathological profiles of MCE clearly indicate its potential antidiabetic activity and other beneficial effects in amelioration of diabetes associated complications. Further, an evaluation of its antilipidemic activity in old obese rats demonstrated significant lowering of cholesterol and triglyceride levels while elevating HDL-cholesterol levels. Also, the extract lowered serum lipids in alloxan diabetic rats, suggesting its usefulness in controlling metabolic alterations associated with diabetes.

## Background

The Indian system of medicine has treated diabetes with its herbals for ages. Vegetables are among the numerous plant adjuncts tried for the treatment of diabetes mellitus. In recent years, there has been a renewed interest to screen such plant food materials, especially, to examine the long-term beneficial effect of dietary vegetables, to identify the active principle, and to understand the mechanism of action, which is at present unclear. Virtually, all forms of diabetes mellitus are caused by a deficiency of insulin secretion or by a combination of insulin resistance and inadequate insulin secretion. Hyperglycemia is the most consistent sign of diabetes, but is not a sensitive indicator at the onset of the disease. GHb, glycosylated haemoglobin is abnormally high in diabetes, with chronic hyperglycemia and often reflects their metabolic control [1]. Liver is an insulin dependent tissue, which plays a pivotal role in glucose and lipid homeostasis and is severely affected during diabetes [2]. During diabetes a profound alteration in the concentration and composition of lipid occurs [3]. Decreased glycolysis, impeded glycogenesis and increased gluconeogenesis are some of the changes of glucose metabolism in the diabetic liver. Diabetes mellitus is known to cause hyperlipidemia through various metabolic derangements. Among several metabolic derangements, insulin deficiency has been known to stimulate lipolysis in the adipose tissue and give rise to hyperlipidemia and fatty liver. Thus, in diabetes hypercholesterolemia and hypertriglyceridemia often occur [4].

This paper describes the study of Momordica *charantia Linn *as an antidiabetic herbal.

*M. charantia *also known as bittermelon or bittergourd belongs to the family Cucurbitaceae. The hypoglycemic activity of *Momordica charantia *has been observed and documented on many occasions [5-8]. Its fruits, leaves and stems have been extensively used and reported for its hypoglycemic effect. Compounds isolated from the fruits & seeds that are believed to contribute to its hypoglycemic activity include charantin (a steroidal glycoside), vicine (a glycoalkaloid) and polypeptide 'p' (a 166 residue insulinomimetic peptide).

*Momordica charantia *has been hypothesized to act via both pancreatic and extra-pancreatic mechanisms [5,6]. Various studies on *M. charantia *have suggested its potential benefit in diabetes. But a lack of proper biomarkers and appropriate parameters for standardization of its preparations has often resulted in varied efficacy and safety. This study was thus initiated with an aim of evaluating the effects of a standardized MCE on blood glucose, serum insulin, serum lipid levels, glucose uptake and glycogenesis in tissues of alloxan diabetic rats. It, thus, investigates the hypoglycemic activity and probable underlying mechanisms of action of the extract by determining changes in various biochemical & histopathological parameters. The study also evaluates the hypolipidemic activity of MCE in alloxan diabetic and old obese rats which would be beneficial in the overall treatment and control of diabetes mellitus.

## Methods

### Animals

Albino rats of the Wistar strain, of either sex, weighing 150–200 g, were purchased from Bharat Sera & vaccines Ltd., Mumbai, India and housed under standard environmental conditions (temperature: 24 ± 1°C, light/dark cycle: 10/14 h). The rats were fed with standard pellet diet (Amrut laboratory animal feed, Maharashtra, India) and water *ad libitum*. Animals described as fasted were deprived of food for 10 h but had free access to water. Animals described as old obese included old male albino Wistar rats 18 months of age weighing 325–350 g, which were maintained on the usual diet during the entire period of study.

The institutional animal house is registered with the Govt. of India and bears the registration No.25/1999/CPCSEA. Experimental protocols were reviewed and approved by the institutional animal ethics committee and conform to the Indian National Science Academy Guidelines for the use and care of experimental animals in research.

### Source of MCE

An authentic standardized MCE was obtained from Amsar Pvt. Ltd, Indore, India. It is an ethanolic extract which has been further fractionated in ethyl acetate and supplied as a 10% processed powder [Lot No: 570, May 2003]. The preparation has been standardised and optimised at the commercial plant of Amsar Pvt. Ltd., Indore, India.

MCE was tested for its acute and short-term toxicity in rats. An acute dose upto 4800 mg/kg was found safe without any abnormal behaviour in rats. Sub-chronic toxicity upto dose of 1000 mg/kg was found safe and did not show any haematological or biochemical alterations in the rats.

### Drugs and chemicals

Alloxan monohydrate was purchased from Loba Chemie, Mumbai, India. Glibenclamide was procured from Aventis Pharma, Mumbai, India. All other chemicals were obtained from local sources and were of analytical grade.

### Preparation of extract, reference drug and alloxan

The extract was administered orally to rats at various doses, as a suspension in 1% w/v CMC, carboxy methyl cellulose. glibenclamide was used as a reference drug and was administered orally at 4 mg/kg as a suspension in 1% w/v CMC. Alloxan was prepared at a concentration of 100 mg/ml in acetate buffer (pH 4.5).

### Experimental procedure

#### Study of the anti-diabetic activity of MCE in hyperglycemic rats

##### Alloxan induced diabetes

Diabetes was induced in rats by a s.c. injection of alloxan monohydrate 100 mg/kg in acetate buffer (pH 4.5). Six days later, blood samples were drawn and tested for blood glucose to confirm diabetes. The diabetic rats showing blood glucose levels above 250 mg% were selected for the following studies.

##### Glucose tolerance curve in alloxan diabetic rats

The diabetic rats were randomly divided into 5 groups of 6 animals each. Three groups received MCE at 150, 300 and 600 mg/kg, p.o. The 4^th ^group was administered glibenclamide (4 mg/kg, p.o.) while the 5^th ^group served as diabetic control, 1% CMC (10 ml/kg, p.o.). All groups received glucose solution (1 g/kg) 30 minutes after the above treatments. Blood levels were determined at 0.5, 1.5 and 2 h after glucose administration.

##### Estimation of biochemical parameters

A group of 32 diabetic rats overnight fasted were randomly divided into 4 groups (8 rats/group) and treated orally with 1% CMC (10 ml/kg), MCE (150 mg/kg and 300 mg/kg) and glibenclamide (4 mg/kg) each, prior to food. Another group of 8 normal healthy rats was used as a normal control. The treatments were continued for 30 days. Effect of MCE (300 mg/kg) and glibenclamide at (4 mg/kg) on normal rats for 30 days was also carried out to see per se effect. Blood glucose levels were determined on different days viz. 0, 7^th^, 14^th^, 21^st ^and 30^th ^day (24 h after the previous dose) by collecting blood from tail vein. On the 31^st ^day, overnight fasted rats were sacrificed and blood was collected for various biochemical estimations. GHb% was determined in heparinised whole blood by ion exchange resin method [9] using commercial kit from Vector Biotek Pvt Ltd, Gujarat, India. Further, the HbA_1c _fraction and mean blood glucose were calculated from the GHb%. Serum insulin was determined by insulin microplate ELISA technique using a commercial kit from Monobind Inc, CA, USA. Total cholesterol was determined in serum using a commercial kit from Biolab Diagnostics, India [10]. Serum triglycerides were determined using a commercial kit from Transasia Bio-Medicals Ltd, India [11]. Total protein was determined in the serum by the method of Lowry et al [12]. The liver was homogenized in 5 % w/v trichloroacetic acid and its glycogen content was determined by the method of Caroll et al [13].

##### Effect on glucose uptake by hemidiaphragm and liver glycogenolysis

The hemidiaphragms and livers of rats treated daily for 30 days as described above were isolated after sacrificing under ether anaesthesia. The tissues were carefully excised and placed immediately in ice cooled perfusion solution with the following composition: NaCl (0.687%), KCl (0.04%), MgSO_4 _(0.014%), CaCl_2 _(0.028%), NaHPO_4 _(0.014%) and NaHCO_3 _(0.21%). Glucose was added to another batch of the perfusate at a concentration of 400 mg%. This perfusate was used to study the glucose uptake/transfer processes. The hemidiaphragms were incubated at 37°C for 1.5 h with appropriate aeration to enable stirring and also to provide oxygen to the tissue. At the end of the incubation period glucose concentration in the perfusate was assayed. The diaphragms were removed, rinsed in water and dried in an oven at 55–60°C for 4–5 h or till a constant weight was obtained. The glucose uptake during the incubation period was calculated in terms of mg/100 mg dry weight of diaphragm. Similarly, liver slices were incubated in the glucose enriched perfusate. The glucose concentration in the perfusate after the incubation period was determined in terms of mg/g of dry weight of liver.

##### Histopathological study of pancreas of alloxan diabetic rats

The pancreas of 4 groups of alloxan diabetic rats treated with MCE (150 mg/kg and 300 mg/kg), glibenclamide (4 mg/kg) and 1% CMC (10 ml/kg) respectively, for a period of 30 days and one group of normal control rats were isolated and preserved in 10%(v/v) formalin. Histopathological evaluation of the tissues [14] was carried out at clinico-pathology laboratory, Haffkine Institute, Mumbai, India. The histological effects were transformed into numerical scoring system and finally expressed in terms of mean ± SEM

##### Evaluation of hypolipidemic activity of MCE in old obese rats

A group of 24 old obese male albino Wistar rats [14] were divided into 3 groups of 8 animals each. The groups received the following treatments daily for 30 days. Group A served as a control and received only 1% w/v CMC suspension. Group B and C received MCE orally at 150 and 300 mg/kg respectively. On the 31^st ^day overnight fasted rats were weighed, sacrificed under ether anesthesia and their blood was collected by cardiac puncture for serum separation. The sera were collected by centrifugation of the clotted blood at 2500 rpm at 30°C for 15 min. The serum samples were used for the determination of total cholesterol, triglyceride and HDL-CH levels using commercial kits as described earlier. Further, LDL-CH and VLDL-CH levels were calculated from the data obtained.

##### Statistical analysis

The results were expressed as Mean ± SEM. The data obtained was subjected to statistical analysis using one-way ANOVA followed by Dunnett's post test for comparison between control and test groups. A '*p*' value < 0.05 was considered to be significant.

## Results

### Studies in diabetic rats

#### a) Glucose tolerance curve in alloxan diabetic rats

**Figure 1:**
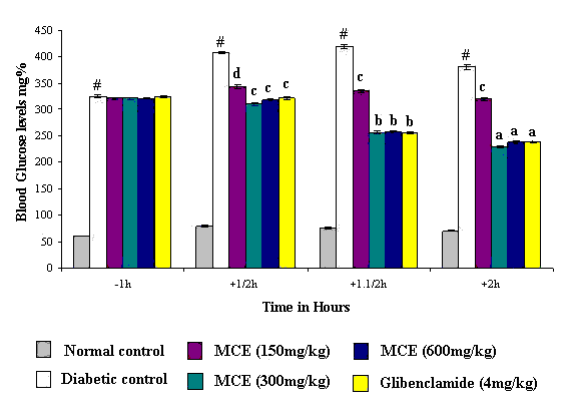
**Effect of Momordica charantia extract on the glucose tolerance of alloxan diabetic rats**. Values are mean ± SEM from 6 animals in each group. Significant difference of diabetic control from normal control :^#^P < 0.001. Significant difference of treated groups from diabetic control on the corresponding time intervals: ^a^P < 0.001, ^b^P < 0.005, ^c^P < 0.01, ^d^P < 0.05.

MCE/glibenclamide treatment significantly inhibited the rise in blood sugar levels in glucose loaded rats. The extract at 300 mg/kg showed an effect equivalent to that of glibenclamide (4 mg/kg). As the inhibitory effect of MCE (600 mg/kg) was comparable to that of 300 mg/kg, it was considered that MCE (300 mg/kg) may be the ceiling dose (Figure 1) for its inhibitory effect.

### b) Estimation of biochemical parameters

Alloxan diabetic rats showed significant elevation in the blood glucose, glycosylated hemoglobin and serum lipid levels while the serum insulin, total protein and liver glycogen levels were decreased significantly in comparison to normal rats. Administration of MCE and glibenclamide restored the above parameters significantly towards normal. The effect of MCE (300 mg/kg) body weight was more significant than that of 150 g/kg and was comparable with that of glibenclamide (4 mg/kg).

**Figure 2:**
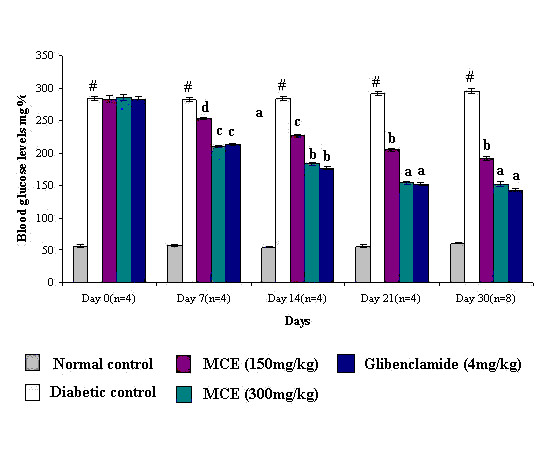
**Effect of Momordica charantia extract on the blood glucose levels of alloxan diabetic rats on various treatment days**. Values are mean ± SEM. N = number of animals in each group. Significant difference of diabetic control from normal control: ^#^P < 0.001. Significant difference of treated groups from diabetic control on the corresponding days: ^a^P < 0.001, ^b^P < 0.005, ^c^P < 0.01, ^d^P < 0.05.

The extract elicited a hypoglycemic effect in alloxan induced diabetic rats as observed by the decrease in blood glucose levels determined on various days during the study (Figure 2). The initial blood glucose levels of the diabetic rats selected for the study were in the range of 260–290 mg%. In the untreated group, the blood glucose further increased to 295.5 mg% on the 30^th ^day. In the diabetic rats treated with MCE (150 mg/kg and 300 mg/kg), the blood glucose levels decreased steadily to 192.0 mg% and 152.4 mg% respectively, on the 30^th ^day. Glibenclamide (4 mg/kg) lowered the blood glucose levels to 142.8 mg% following a 30 day treatment. The effect of MCE (300 mg/kg) was comparable to that of glibenclamide while that of MCE (150 mg/kg) was lower.

**Figure 3:**
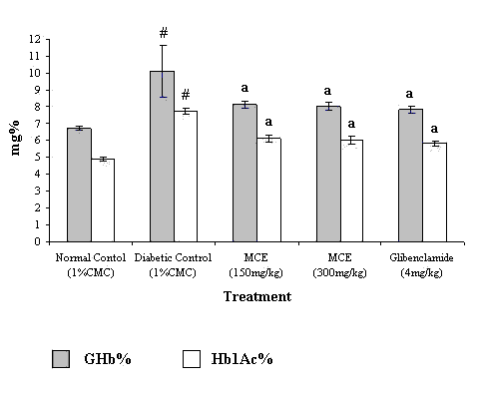
**Effect of Momordica charantia extract on glycosylated hemoglobin levels (GHb%) and HbA1c fraction of alloxan diabetic rats**. Values are mean ± SEM from 8 animals in each group. Significant difference of diabetic control from normal control: ^#^P < 0.001. Significant difference of treated groups from diabetic control on the corresponding days: ^a^P < 0.001.

Treatment with MCE reduced percent GHb levels from 10.1% (in diabetic control) to 8.0% and 8.1% in rats treated with 300 mg/kg and 150 mg/kg doses respectively. The GHb levels were found to be 7.8% in glibenclamide 4 mg/kg treated rats (Figure 3).

Insulin levels were found to be much lower (1.6 μIU/ml) in the alloxan diabetic untreated rats when compared with the nondiabetic control (3.5 μIU/ml) group. Treatment with MCE as well as glibenclamide significantly elevated the insulin levels (Table 1).

**Table 1:** Effect of *Momordica charantia *extract on the serum insulin, cholesterol, triglyceride and total protein levels of alloxan diabetic rats

Oral treatment	Insulin μIU/ml (n = 6)	Cholesterol mg% (n = 8)	Triglycerides mg % (n = 8)	Protein g% (n = 8)
	
	Mean ± SEM			
Normal control	3.5 ± 0.4	78.8 ± 0.8	58.3 ± 0.4	7.7 ± 0.1
Diabetic control	1.6 ± 0.2^#^	109.6 ± 1.2^#^	117.1 ± 2.8^#^	6.0 ± 0.1^#^
MCE (150 mg/kg)	2.2 ± 0.3	92.2 ± 2.3^a^	100.3 ± 1.6^a^	7.1 ± 0.2^a^
MCE (300 mg/kg)	2.6 ± 0.3^c^	78.0 ± 1.6^a^	97.8 ± 2.6^a^	7.4 ± 0.4^a^
Glibenclamide (4 mg/kg)	2.7 ± 0.2^a^	81.5 ± 1.0^a^	93.2 ± 2.3^a^	7.5 ± 0.3^a^

Significant difference of diabetic control from normal control : ^#^P < 0.001

Significant difference of treated groups from diabetic control on the corresponding days: ^a^P < 0.001, ^c^P < 0.01.

The diabetic rats showed elevated levels of serum cholesterol, triglycerides and decreased total protein levels. Following treatment with MCE (300 mg/kg) or glibenclamide (4 mg/kg), cholesterol, triglyerides and protein levels were restored to nearly normal (Table 1). There was a marked reduction in the liver glycogen levels of alloxan diabetic rats from 3.8 g/100 g tissue (in normal control rats) to 0.84 g/100 g tissue. MCE (150 mg/kg) treatment showed 72.6% increase while at 300 mg/kg there was 100% increase in liver glycogen levels as compared with the untreated diabetic rats. Glibenclamide treatment elicited 98.8% increase in liver glycogen levels when compared to the untreated diabetic rats (Table 2).

**Table 2:** Effect of *Momordica charantia *extract on liver glycogen levels, glucose uptake by hemidiaphragm and glycogenolysis by liver of alloxan diabetic rats

Oral treatment (n = 8)	Liver glycogen g/100 g	Glucose uptake by hemidiaphragm mg/100 mg	Glucose transfer by liver mg/g	Pancreas histoarchetechture necrotic changes & persistence of islets of Langerhans*
	
	Mean ± SEM			
Normal control	3.8 ± 0.4	16.0 ± 0.4	26.9 ± 0.4	0.3 ± 0.01
Diabetic control	1.0 ± 0.2^#^	4.7 ± 0.2^#^	42.6 ± 2.2^#^	3.6 ± 0.13
MCE (150 mg/kg)	1.6 ± 0.2^b^	8.1 ± 0.3^a^	17.9 ± 0.4^a^	2.9 ± 0.2
MCE (300 mg/kg)	1.7 ± 0.1^a^	15.9 ± 0.4^a^	15.6 ± 0.7^a^	1.9 ± 0.12
Glibenclamide 4 mg/kg	1.8 ± 0.1^a^	14.0± 0.3^a^	17.9 ± 0.8^a^	1.8 ± 0.18

Significant difference of diabetic control from normal control: ^#^P < 0.001

Significant difference of treated groups from diabetic control: ^a^P < 0.001, ^b^P < 0.005,

*Scoring system: 0 – Normal, 1 – very mild, 2 – mild, 3 – moderate, 4 – marked

Treatment with MCE (300 mg/kg) and glibenclamide (4 mg/kg) for 30 days elicited insignificant changes in all biochemical parameters assayed. Hence results are not shown in the table.

#### c) Effect on glucose uptake by hemidiaphragm and glycogenolysis by the liver

Hemidiaphragms taken from rats treated with MCE and glibenclamide showed a significant enhancement of the glucose uptake process as compared to diabetic untreated rats. Also, both MCE and glibenclamide treated rats showed a significant inhibitory effect on glycogenolysis in liver slices (Table 2).

#### d) Histopathological study of the pancreas of alloxan diabetic rats

The normal control rats showed no architectural changes in the histology of the pancreas. In the alloxan diabetic untreated rats, the islets of Langerhans showed diffused necrotic changes of moderate to marked degree as a result of which they were significantly reduced in size and number. Only occasional presence of the islets could be detected in a few rats. The group of rats treated with glibenclamide showed diffused necrotic changes of mild to moderate degree in the pancreas. There was a mild reduction in the size and number of the islets in this group. The effect of MCE (150 mg/kg) on alloxan diabetic rats was comparable with that of glibenclamide. The MCE (300 mg/kg) treated group of rats showed mild to moderate degree of necrosis of the islets of Langerhans. The pancreatic damage observed in glibenclamide and MCE (150 mg/kg) treated diabetic animals was milder than that found in the untreated diabetic control group. Pancreas of MCE (300 mg/kg) treated rats showed milder damage in comparison to that observed in the glibenclamide and MCE (150 mg/kg) treatment groups. (Table 2).

### Hypolipidemic effect of MCE in old obese rats

The old obese rats selected for the study had elevated serum lipid levels. Following 30 days treatment with MCE at 150 & 300 mg/kg doses, the rats showed dose dependent reduction in cholesterol, triglyceride, LDL-CH, VLDL-CH and blood glucose levels as compared to untreated control. Also, MCE caused elevation of serum HDL-CH levels (Table 3).

**Table 3:** Effect of MCE on the serum lipids and blood glucose levels in old obese rats

Oral treatment (n = 8)	Cholesterol (mg %)	HDL-CH (mg %)	VLDL-CH (mg %)	LDL-CH (mg %)	Triglyceride (mg %)	Blood glucose (mg %)
	Mean ± SEM					
Normal control	80.8 ± 1.7	18.7 ± 0.9	8.4 ± 0.	54.4 ± 0.21	60.4 ± 0.39	68.8 ± 2.1
Obese control	115.9 ± 2.7^b^	27.8 ± 0.4^a^	24.6 ± 0.8^b^	64.4 ± 2.2^a^	123.2 ± 4.1^b^	116.5 ± 1.6^b^
MCE (150 mg/kg)	88.5 ± 1.7^c^	31.2 ± 0.6^c^	20.2 ± 0.9^c^	37.2 ± 2.0^d^	100.9 ± 4.4^c^	94.5 ± 1.1^a^
MCE (300 mg/kg)	81.6 ± 1.9^d^	32.4 ± 0.6^d^	17.8 ± 0.2^d^	26.3 ± 3.1^d^	88.7 ± 3.2^d^	77.1 ± 1.2^d^

Significant difference of Obese control from Normal control: ^a^P < 0.05, ^b^P < 0.01,

Significant difference of treatment groups from Obese control: ^c^P < 0.01, ^d^P < 0.001

## Discussion

Alloxan is known for its selective pancreatic islet β-cell cytotoxicity and has been extensively used to induce diabetes mellitus in animals. In our present experimental studies it was observed that MCE can reverse the metabolic derangements occuring in alloxan induced diabetes in rats.

To gain an understanding of the mechanism(s) by which MCE elicits its hypoglycemic activity, various biochemical parameters were evaluated following subchronic (30 day) treatment in rats. Our experimental findings suggest a significant reduction in i) blood glucose levels on different days, ii) glycosylated hemoglobin levels and its HbA_1c _fraction and iii) serum cholesterol and triglyceride levels in both, MCE and glibenclamide treated rats. MCE and glibenclamide elevated the reduced serum insulin, total protein and liver glycogen levels. Glycosylated peptides are elevated several fold in diabetes. Since the average life span of a red blood cell is 120 days, the assumption of the clinical importance of GHbA levels is that they represent time-averaged values for blood glucose over the last or next 3 to 4 month period. This provides a simple, useful means of assessing treatment efficacy and patient compliance. The increase in serum insulin levels suggested that MCE like glibenclamide enhances the secretion of insulin from the beta cells of the islets of Langerhans. Further, it has an ability to restore the protein breakdown and enhance the glycogenesis process in the liver of diabetic rats. In addition, *in vitro *tests indicated that MCE and glibenclamide can induce stimulation of the glucose uptake process by diaphragm and inhibition of the glycogenolysis in liver of rats. Histopathological studies revealed that MCE and glibenclamide significantly improved the histological architecture of the islets of Langerhans. The groups treated with MCE (150 and 300 mg/kg) and glibenclamide (4 mg/kg) showed greater persistence of the islets of Langerhans and lesser degree of necrotic changes as compared to the untreated alloxan diabetic rats.

Hence, tentatively, it may be summarized that the possible mechanism(s) by which MCE brings about its antihyperglycemic action may be through potentiation of pancreatic secretion of insulin from the intact β-cells of islets (which was clearly evidenced by the increased level of insulin in diabetic rats treated with MCE and glibenclamide) coupled with extra-pancreatic mechanisms like decreased glycogenolysis and enhanced glycogenesis by the liver and/or enhanced transport of blood glucose to peripheral tissues (as seen by the stimulatory effect on glucose uptake in rat diaphragm). MCE's direct effect on the regeneration of the islets of pancreas was also evidenced by the restoration of the architecture of the islets of Langerhans in histopathological studies. With such an evidence, it is possible to assume that MCE might stimulate the secretion of insulin from the beta cells by a mechanism similar to that of oral hypoglycemic agents (like sulfonylureas) i.e. by depolarization of islet membrane which consequently alters the change in ion flux [15] or affecting receptors responsible for the recognition of insulin secretagogues [16]. These mechanism(s) have been accepted as a paradigm for the action of all insulin releasing agents.

Hypercholesterolemia and hypertriglyceridemia have been reported to occur in alloxan diabetic rats [17,18]. A significant increase in serum cholesterol and triglycerides observed in our experiment is in agreement with the findings of the aforementioned authors. The marked hyperlipidemia that characterizes the diabetic state may therefore be regarded as a consequence of the uninhibited actions of lipolytic hormones on the fat depots [4]. MCE treatment to old obese rats elicited a dose related hypolipidemic activity. All the lipid components viz cholesterol, LDL-CH and triglycerides were reduced significantly. The more prominent effect being reduction in LDL-CH which is a known triggering factor for coronary occlusion or its block. Similarly HDL-CH is a protective cholesterol and responsible for transportation of cholesterol. Considering MCE's effect on these lipid components, it can be assumed a potential hypolipidemic agent, which will be a great advantage both in diabetic condition as well as the associated atherosclerosis or hyperlipidemic conditions.

The antihyperlipidaemic effect of MCE may be due to the down regulation of NADPH and NADH cofactors in the fat metabolism. MCE may exert its antilipidemic action by oxidizing NADPH.

## Conclusion

From our experimental findings it is possible to conclude that MCE exhibited promising antidiabetic activity in alloxan diabetic rats. Its antihyperlipidemic effect could represent a protective mechanism against the development of atherosclerosis, especially in diabetic condition. Finally, it can be considered that MCE is safe for oral consumption and elicits promising hypoglycemic activity in animal experiments. Hence, it may be pursued for its clinical usefulness in the management of diabetes mellitus and other associated complications.

## Abbreviations

MCE, *Momordica charnatia *Extract, GHb, Glycosylated haemoglobin

## Competing interests

The author(s) declare that they have no competing interests.

## Authors' contributions

**SRN **conceived, designed, co-ordinated and supervised the study and the writing of the manuscript. **NPF **initiated the study, carried out the experimental and performed statistical analysis. **CVL **and **VSP **aided **NPF **in the study and helped to draft the manuscript. All authors read and approved the final manuscript.

## Pre-publication history

The pre-publication history for this paper can be accessed here:


